# An Exposition of the Guild Rules

**Published:** 1913-07-26

**Authors:** 


### The National Medical Guild.

### An Exposition of the Guild Rules (*continued*).

Last week Rule 2 was under consideration, and there remain two or three lines which have not yet been considered.

### Amalgamation.

Powers to amalgamate are taken in the words "to federate or amalgamate with any other body or society." A trade union may federate with almost any body of whatever nature, and it does not imply more than a certain amount of mutual politeness. It may mean a common decision on some burning question of the day, but the one thing which it cannot mean is any common fund. A trade union could presumably federate with the British Medical Association—if that body so desired.

Amalgamation is a more serious matter, for it means that the amalgamating bodies for all practical purposes become one—the identity of each is merged in the new body. In particular there is financial unity; like the early Christians, they have all things in common. They would have a common board of directors or Council—whichever term was preferred —and a common subscription.

### Premises.

It will be noted that the last part of Rule 2 is as follows: "To acquire, lease, or build premises for the purposes of the Guild." The law is rather strict on this point, and it would appear that an unregistered trade union cannot build or permanently acquire premises in such a way that they become the absolute property of the union. A registered trade union (the National Medical Guild is registered) may do so if the buildings do not cover more than one acre—buildings used by branches do not count towards the acre. No doubt the intention of the law was to prevent unions acquiring house property of any considerable extent or of such a nature that it made a profit from rents. A trade union is not supposed to trade for profit (a limited company usually exists solely for this purpose, and has the appropriate rights), and hence any property which might be a source of income is forbidden them.

### Rule 3.—Membership.

Membership in the Guild shall be by election in accordance with Rule 4 hereof. Provided always, except in the case of honorary members, no one shall be entitled to become a member unless he is a duly qualified medical practitioner as defined by Section 34 of the Medical Act, 1858.

Honorary members are provided for in accordance with a very general rule of all societies, but the Guild has not at present any intention of electing any. It is a rule made in case such a measure should be deemed desirable.

The sort of individual who might be made an honorary member would perhaps have rendered some signal service to the profession or the Guild in some dispute, or the Guild might elect as honorary members medical men whose practice was of such an ultra-respectable nature that the weal or woe of the profession at large did not touch them, but who yet desired to assist their fellows by their interest and, possibly, by donations. As a matter of fact, the Guild is already indebted to various consultants for donations.

The Guild does not include members of the dental profession, except they have also a medical qualification. At present this section of medical practitioners has not been attacked or asked to work under a scheme of which they have not a high opinion; on the other hand, they are very much exposed to the competition of unqualified practitioners. There are signs that they will be soon involved, since dental benefit is freely spoken of as one of the "additional benefits" which societies ought to offer as soon as may be. Such "additional benefits" are not to be confused with the benefits conferred on dependants of the insured when they shall be brought under some extension of the Act. These would be benefits to additional persons, but not additional benefits.

### Rule 4.—Election of Members.

Anyone desiring to become a member of the Guild must be proposed and seconded by two members who have a personal knowledge of such person. The name of the proposed new member, together with the names of the proposer and seconder, shall be submitted to the Council at its next meeting, when a ballot shall be taken; and if a majority of the Council present vote in his favour he shall be held to be elected a member of the Guild. Provided always, when the proposed member has already been a member of the Guild and for some reason he has ceased to be a member, the Council may make it a condition of his membership that he shall pay a sum of money equivalent to what he would have paid up to that date by way of contributions and levies if he had remained a member of the Guild continuously, dating from the time when his membership ended; and if at the time at which his membership ended he was in arrear with his contributions and/or levies, such arrears and/or levies may be added to the sum charged; but it shall always be open for the Council to impose a lesser sum as a condition of membership.

The first lines of this rule deal with the proposing and seconding of members "by members who have a personal knowledge" of the applicants for membership. It is obvious that if this were strictly enforced members could only be obtained with the greatest difficulty during the youth of the Guild. Hence the first part of this rule is waived for the present; it is sufficient for an intending member to send his name and subscription to the office at 34 Villiers Street, W.C.

The latter part of the rule is rather involved, but the meaning is easily deduced from the necessary verbiage. It is to the effect that a member cannot cease to be a member in order to avoid subscriptions and then rejoin as if he had never belonged before, thus obtaining all the benefits that others have paid in for during the time that he has been a defaulter. On the contrary, he will have to make good the subscriptions for the time during which he was a non-member.

There is a saving clause whereby the Council may reduce the sum claimed, *e.g.* in the case of a practitioner who practised abroad for several years and only returned to this country later, but who had been a member before he left for other climes. In certain cases it may also be necessary to make a reduction where arrears have been necessitated by financial circumstances. It is not, and never has been, the intention of the Guild to exclude the less favourably situated members of the profession on the sole ground that financial circumstances do not allow them to keep up subscriptions or, in particular, levies; but, on the other hand, mere slackness cannot be accepted as an excuse for non-payment. Such slackness will certainly exist, and we shall find members paying when there is trouble and falling off when there is no trouble. For such there can be no mercy, and it would be grossly unfair to the constant members if those in arrear were allowed to have the same benefits for a less payment.

It will be noted that elections are in the hands of the Council, but it may be mentioned that it is not the intention of the Council to oppose the wishes of local branches in this respect.

Rule 5.—Vice-Presidents.

The Council may annually at the meeting of the Council held next after the annual meeting elect such person or persons as any member or members of the Council may choose to nominate as vice-presidents of the Guild.

The question of honorary members has already been dealt with. Such vice-presidents as are here mentioned are not to be confused with the deputy-president or his office. The latter has a vote on the Council, and presides in the absence of the president, but vice-presidents have no vote nor any seat on the Council or share in the management of the Guild.

### *Guild Notes.*

Brighton.

This Number will appear during the annual meeting of the British Medical Association at Brighton. It has already been announced that the Guild is opening an office there.

The decisions of the British Medical Association will have been made before this appears, but too late for announcement here. Next week we shall have something to say on the matter, and especially concerning the position of the National Medical Guild in relation to those decisions. It may, therefore, be necessary to hold over consideration of the rules for a week or two.

Approved Societies.

The Guild has lately had several cases brought to its notice in which the patients of members have been threatened with expulsion from their societies on the grounds of false statements in their forms of application. Thus a woman who claimed for sick pay put in a certificate from a specialist to the effect that she was suffering from a congenital specific lesion. This had not shown itself up to the time that she first consulted a doctor, and she had no idea that she had any congenital taint. Nevertheless the society threatened her with expulsion, and refused sickness benefit.

It may interest members to know that a society must prove "wilful" misstatement if the expulsion is in order. It is apparent that in the case given this was impossible. The Guild will be pleased to advise members whose patients have appealed to them in connection with such cases as to the steps which the patients should take.

